# Supplementary material for: Seminoma and Embryonal Carcinoma Footprints Identified by Analysis of Integrated Genome-Wide Epigenetic and Expression Profiles of Germ Cell Cancer Cell Lines
Source: PLoS One. 2014 Jun 2;9(6):e98330. doi: 10.1371/journal.pone.0098330 (PMC4041891; doi:10.1371/journal.pone.0098330)
Supplement: File S1 — ZIP file containing DMRforPairs output for significant regions. Please start from the html files. (ZIP) [file pone.0098330.s008.zip › figures/14586.pdf]

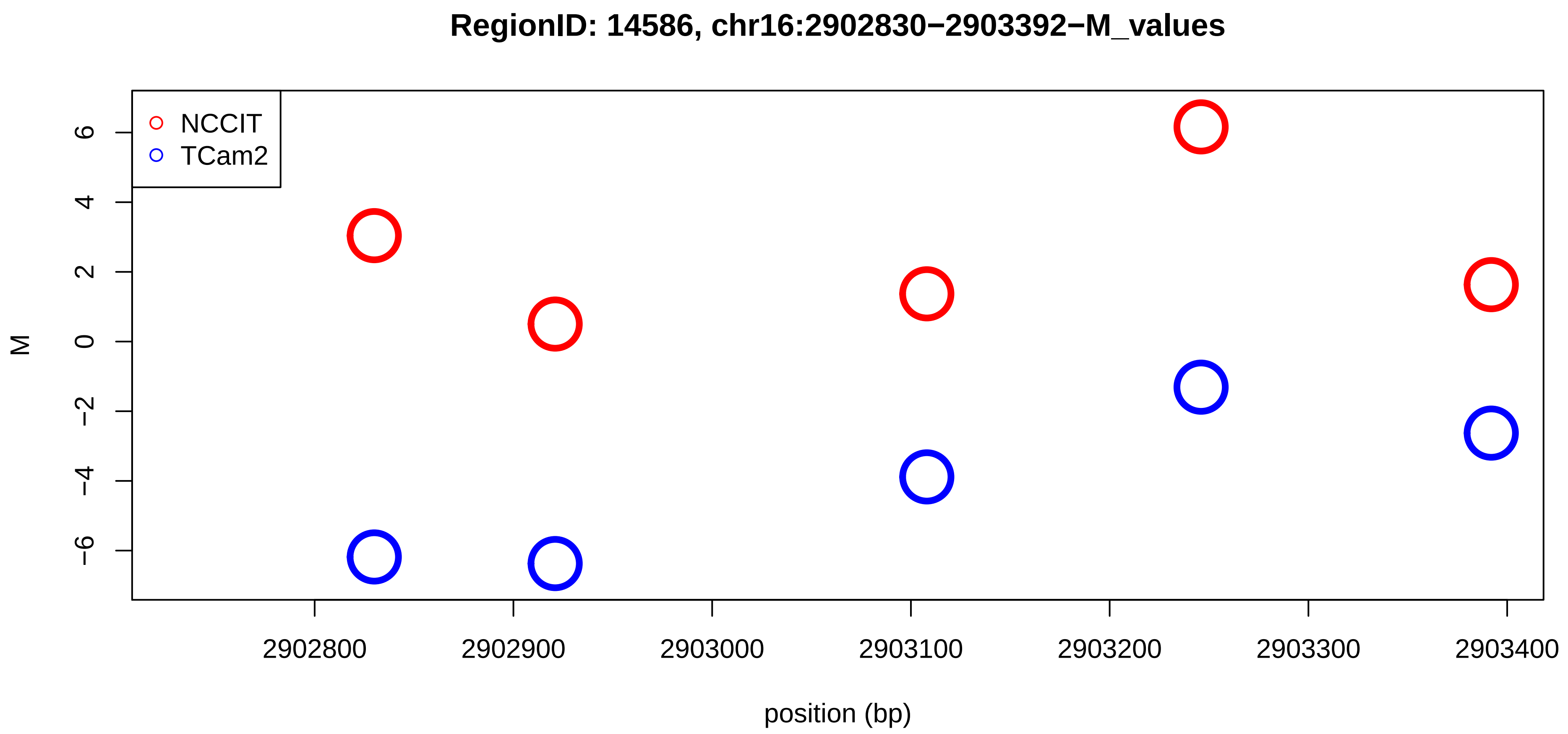
RegionID: 14586, chr16:2902830−2903392−M_values
NCCIT
TCam2
M
6
4
2
0
−2
−4
−6
2902800
2902900
2903000
2903100
2903200
2903300
2903400
position (bp)

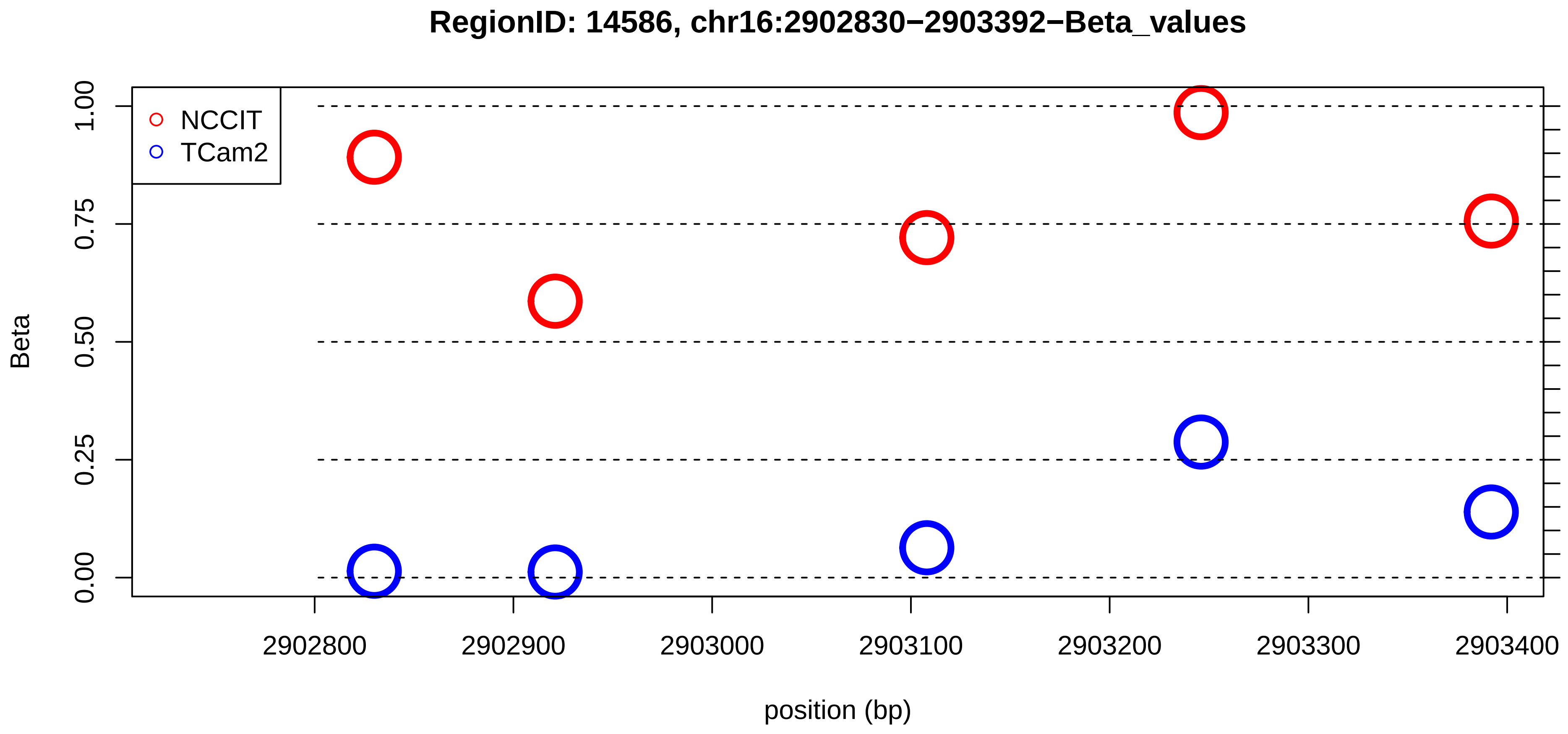
RegionID: 14586, chr16:2902830−2903392−Beta_values
NCCIT
TCam2
Beta
1.00
0.75
0.50
0.25
0.00
2902800
2902900
2903000
2903100
2903200
2903300
2903400
position (bp)
